# Multi-country collaboration in responding to global infectious disease threats: lessons for Europe from the COVID-19 pandemic

**DOI:** 10.1016/j.lanepe.2021.100221

**Published:** 2021-10-07

**Authors:** Mark Jit, Aparna Ananthakrishnan, Martin McKee, Olivier J. Wouters, Philippe Beutels, Yot Teerawattananon

**Affiliations:** a Department of Infectious Disease Epidemiology, Faculty of Epidemiology and Population Health, London School of Hygiene & Tropical Medicine, London, UK; b School of Public Health, University of Hong Kong, Hong Kong SAR, China; c Health Intervention and Technology Assessment Program, Ministry of Public Health, Thailand; d Department of Health Services and Policy, Faculty of Public Health and Policy, London School of Hygiene & Tropical Medicine, London, UK; e Department of Health Policy, London School of Economics and Political Science, London, UK; f Centre for Health Economic Research and Modelling Infectious Diseases, Vaccine & Infectious Diseases Institute, University of Antwerp, Antwerp, Belgium; g School of Public health and Community Medicine, University of New South Wales, Sydney, Australia; h Saw Swee Hock School of Public Health, National University of Singapore, Singapore

## Abstract

Since 2005, the world has faced several public health emergencies of international concern arising from infectious disease outbreaks. Of these, the COVID-19 pandemic has had by far the greatest health and economic consequences. During these emergencies, responses taken by one country often have an impact on other countries. The implication is that coordination between countries is likely to achieve better outcomes, individually and collectively, than each country independently pursuing its own self-interest. During the COVID-19 pandemic, gaps in multilateral cooperation on research and information sharing, vaccine development and deployment, and travel policies have hampered the speed and equity of global recovery. In this Health Policy article, we explore how multilateral collaboration between countries is crucial to successful responses to public health emergencies linked to infectious disease outbreaks. Responding to future global infectious disease threats and other health emergencies will require the creation of stronger mechanisms for multilateral collaboration before they arise. A change to the governance of multilateral institutions is a logical next step, with a focus on providing equal ownership and leadership opportunities to all member countries. Europe can be an example and advocate for stronger and better governed multilateral institutions.

## 1 Introduction

In 2005, the World Health Organization (WHO) revised its International Health Regulations, taking account of lessons from the Severe Acute Respiratory Syndrome (SARS) pandemic of 2002-3. Henceforth, WHO could declare a public health emergency of international concern (PHEIC) and has since done so six times: the H1N1 influenza pandemic (2009), twice for Ebola outbreaks in several parts of Africa (2014 and 2018), the ongoing polio epidemic (2014), Zika (2015), and COVID-19 (2020). The last of these has had by far the largest global health and economic impact. Since 2005, we have also witnessed the re-emergence or rapid spread of established infectious diseases like dengue, cholera, and measles.

These outbreaks have been characterised by the way that actions taken by one country have had substantial consequences for others [1]. This suggests that international coordination when responding to such outbreaks brings greater overall benefits than each country independently pursuing its own self-interest.

In this Health Policy article, we examine the case for multilateral collaboration on threats from infectious disease. While recognising that there are many perspectives we could adopt, including those from the international relations literature, we begin with the economic case for collaboration, looking at two types of goods with substantial inter-country effects: shared knowledge and population immunity. Next, we explore the case for one type of international collaboration, multilateralism, as a means to achieve an optimal outcome. We then examine its application in several areas: research and data sharing [2], vaccine development and distribution [3], and international travel [4]. We use a scorecard to show the strengths and weaknesses in the global response to the COVID-19 pandemic (Table 1), and draw lessons for future multi-country collaboration, particularly across Europe.

**Table 1:** Scorecard showing global successes and shortcomings in the multilateral response to the COVID-19 pandemic.

Domain	Successes	Shortcomings
Research collaboration and information sharing	• Sharing of information by researchers • International research collaborations • Public data repositories	• Many regions and countries slow to learn policy lessons from elsewhere • Lack of systemic global research governance • Duplication of research studies
Vaccine discovery and development	• Multinational initiatives to fund efforts such as the Coronavirus Global Response and the Coalition for Epidemic Preparedness Innovations • Approval of vaccines and adjuvants • Establishing the principle of equitable vaccine distribution through the COVAX Facility (despite failures in implementation)	• Most funding from national efforts • Most vaccine doses secured by rich countries through bilateral deals • Trade barriers around vaccines and raw materials
Travel policies	• Travel restrictions delayed spread from China in early 2020	• Dissonant COVID-19 response policies between highly connected nations • Restrictions on travel to countries of high COVID-19 incidence contribute little to control in these countries

## 2 Externalities and global public goods

An economic case for action can be built on the principles of externalities and public goods. An externality is a positive or negative effect that falls on one party due to the action taken by another. For instance, someone whose actions lead to contracting an infection may impose negative externalities on the person's close contacts by transmitting the infection to them. Externalities are associated with market failure; the externalities of one person's consumption of a good will not be reflected accurately in the price they are willing to pay for it. Hence externalities are often used to justify government intervention, such as taxes, subsidies, and regulations. When those affected live in different countries, these are cross-border externalities, requiring international coordination to correct market failures. For instance, consumption of COVID-19 vaccines in one country may prevent the emergence of variants that could cause disease elsewhere.

Externalities are often associated with goods that are non-excludable, i.e. if some people are able to consume those goods, then others cannot be prevented from doing so. If a good is non-excludable, then producing it generates positive externalities (increasing its supply to everybody) while consuming it generates negative externalities (decreasing its supply to everybody). However, negative externalities in consumption are not generated if the good is also non-rivalrous, i.e. it can be used by everyone without reducing its availability to others. Goods that are both non-excludable and non-rivalrous are called public goods, and if everyone in the world has access to them, they are global public goods. A classic example of a public good is street lighting.

Emerging infectious diseases are associated with two important global public goods—shared knowledge and population immunity:

***Shared knowledge.*** Information about an emerging infection is crucial to enable countries to prepare and mount public health responses, particularly in the early days of an outbreak [5]. As prevention and treatment technologies are developed, knowledge about their safety, efficacy and production mechanisms brings benefits globally. However, not all countries in the world have surveillance and data analytic infrastructure to collect all these data. Indeed, a single country as the sole unit of analysis is often insufficient to establish which measures are effective during a global health crisis. Pooling resources across countries could enable the most important information to be generated and shared as early as possible. This knowledge is non-rivalrous but potentially excludable: people who have knowledge can still withhold it from others through non-disclosure or intellectual property barriers. If the knowledge is instead shared or made available to all, then it becomes a global public good [6].

***Population immunity.*** If one country controls infection through vaccination, testing/quarantine, or non-pharmaceutical interventions, this is likely to reduce infections elsewhere. This may also curtail the emergence of variants that are more transmissible, cause more severe disease, and/or can evade natural or vaccine-induced immunity [7]. Countries with good infection control may be able to relax non-pharmaceutical interventions, which can stimulate production, demand and international trade. If a country has strong travel connections with other countries that have higher infection prevalence, it may try to prevent disease spread through travel restrictions, but restrictions that are sufficiently stringent to reduce infection spread may impact negatively on economic growth [8].

## 3 The case for multilateralism

Countries can collaborate in different ways. The simplest arrangement is bilateral, where one country reaches an agreement with another, often a neighbour. This is appropriate where the subject of the agreement is highly specific, such as the management of traffic on a waterway they share. However, when multiple countries are involved, as with trade in particular goods, it is inefficient as each country must invest in complex negotiations with many others, replicating the process each time. More often, groups of countries will seek to reach multilateral agreements [9]. These can be topic specific, such as trade or the environment, or more general.

Multilateral relations have three characteristics. First, they are indivisible. All parties are treated equally. For example, outside certain arrangements such as those provided for by the European Union (EU), each member state of the World Trade Organisation must offer the same terms to all others. Second, there is an expectation of diffuse reciprocity. In other words, while one party may derive greater benefit than the other in a particular transaction, these will even out over time. Third, there is some form of dispute resolution to ensure compliance with what has been agreed.

The growth of multilateral agreements since the Second World War testifies to their perceived value, both to small countries that can achieve greater influence by working together and to large countries that often have the outsized influence on rule-setting. However, multilateral agreements involve some pooling of sovereignty, which may elicit opposition from some national politicians. However, in a world that is increasingly governed by multilateral agreements, the cost of being outside the system, in terms of access to the global economy, can be high. In those areas where the cost of unilateral action is greatest, such as nuclear or chemical weapons proliferation, most but not all governments have been willing to accept the benefits of pooling their sovereignty.

The situation has become more complicated in recent decades by the growing power of nonstate actors, some of which have greater power than many nation-states. They exist both in the commercial sector (e.g. pharmaceutical corporations) and in the philanthropic sector (e.g. the Bill & Melinda Gates Foundation). A number of public-private partnerships have emerged, such as Gavi, the Vaccine Alliance.

The advantages of multilateral cooperation are evident but often difficult to achieve. Frieden and colleagues [10] identified four reasons for this difficulty, related to the production of global public goods: (i) creating a shared vision of global public goods is challenging and requires cooperation; (ii) governments face complex domestic challenges, leaving them little capacity to address longer-term global commitments to produce global public goods; (iii) different governments pursue different objectives; e.g. they may share the goal of combating a pandemic but differ substantially in their security, financial, or industrial goals; and (iv) previous failed initiatives have left governments sceptical about the ability of the international community to deliver global public goods.

The COVID-19 pandemic has highlighted weaknesses in the existing multilateral system, set out in an independent panel report [11]. The initial pandemic response was slow, reflecting failings in the application of the revised International Health Regulations and lack of urgency by both countries and WHO. Global leadership coordination was inadequate, and there was lack of investment in pandemic preparedness. A PHEIC declaration did not directly lead to the provision of the financial resources needed. These failures suggest that new mechanisms may be needed to complement existing pandemic response structures, perhaps based on the measures taken by the G20 in the aftermath of the 2009 global financial crisis. There is also scope to harmonise international practice, such as research standards, through agreements between civil society organisations and other non-state actors. An example is the development of global requirements for phase 3 trials of medicines and vaccines under the umbrella of the International Coalition of Medicines Regulatory Authorities [12]. Looking ahead, it is likely that there will be several changes to the global health architecture, possibly including a new pandemic treaty and additional international collaborative mechanisms to promote preparedness and coordinate responses. In the following sections, we explore what those developments might look like in three key areas.

## 4 Research collaboration and information sharing

International collaboration around research on COVID-19 and similar threats has been characterised by an interplay between “scientific globalism” (seeking open and collaborative science to advance knowledge and address joint challenges) and “scientific nationalism” (seeking local scientific advancement to enhance national agendas such as economic competitiveness and foreign policy goals) [13,14]. From the “globalism” perspective, the COVID-19 pandemic saw calls for closer multi-country collaboration to expedite data sharing [15], [16], [17]. Indeed, the international research community – including academic, health, industry, and professional groups – collaborated from the outset, through early exchange of laboratory and surveillance data, genome sequences, and information on clinical outcomes [18,19]. A bibliometric analysis found that the proportion of scientific articles published in the first five months of 2020 that were international collaborations was higher than the historical average [14]. Such collaboration was possible even between researchers in countries where political cooperation was strained, such as America and China [20,21], although it has more recently been threatened by arguments over the origins of SARS-CoV-2 [22]. Despite these examples of success, there is much more that could have been done in several key areas as discussed below:

***Collaborative research.*** Gaps in international research collaboration have led to duplication of efforts. While there could be value in diverse approaches, multiple trials with similar conditions have been conducted on the efficacy of products such as hydroxychloroquine even after they were shown to be ineffective [23,24]. There have been successes, such as the WHO Solidarity Trial, which includes over 30 countries [25], but it had to overcome numerous administrative hurdles in each country. At the same time there have been fewer empirical studies exploring the effectiveness of interventions of immediate policy relevance such as face coverings and testing, quarantine and isolation protocols [24], although some trials of interventions to increase uptake of protective measures such as face coverings have been conducted. International differences in policies also offer great scope for natural experiments, such as through the econometric studies described below.

Major research funders could convene a process to facilitate advanced agreement on generic protocols and streamline ethics and regulatory approvals. Such a process might also explore the scope for greater international harmonisation of such processes outside an emergency. Multinational clinical trials regulation (such as that developed by the EU) could help circumvent redundancies and obstacles [26].

***International exchange of experiences.*** During COVID-19, there has been inadequate learning from other countries’ experiences. In some cases, policymakers may simply have been unaware of what was being done elsewhere. This has been addressed by several regional initiatives, such as the COVID Health System Response Monitor created by the European Observatory on Health Systems and Policies, which collates information on policies adopted across the WHO European Region [27]. Policy response in different countries are now being tracked [28], and econometric studies have been conducted to examine their impact on transmission [29,30].

***Data sharing****.* Public repositories have been created to present comparable indicators of the COVID-19 pandemic, such as cases by country and genomic data on variants. However, these resources are only as good as data provided by countries. Many countries require investment in disease surveillance infrastructure, including vital registration and laboratories. Here, donors and development agencies can play a role, ideally in a coordinated manner to avoid creating incompatible systems. However, countries may be less enthusiastic about sharing information if they are denied access to the fruit of their contributions. For example, WHO shared avian influenza virus sequencing data received from Indonesia to a pharmaceutical company, which then sold the vaccine to target this sequence back to the country. Indonesia subsequently decided to suspend information sharing with the WHO [31]. It is therefore important to incentivise all countries to participate in data sharing by ensuring equitable access to life-saving technologies in exchange for expanded global surveillance knowledge.

Innovation economics offers valuable insights on the benefits of knowledge interchange [32], although so far it has received more attention in discussions on national rather than international development. This field sees knowledge, rather than the more traditional accumulation of capital, as a primary driver of economic growth. This is borne out by empirical analyses, such as the growth of the German biotech sector, from which one of the COVID-19 vaccines emerged [33]. The literature on innovation economics emphasises the importance of tacit knowledge, regimes and policies that encourage entrepreneurship and innovation, technological spillovers between collaborative firms, and systems of innovation that create innovative environments. Despite the economic case however, multilateral collaboration faces political challenges described by Frieden and colleagues [10].

## 5 Vaccine development and distribution

**Figure 1:**
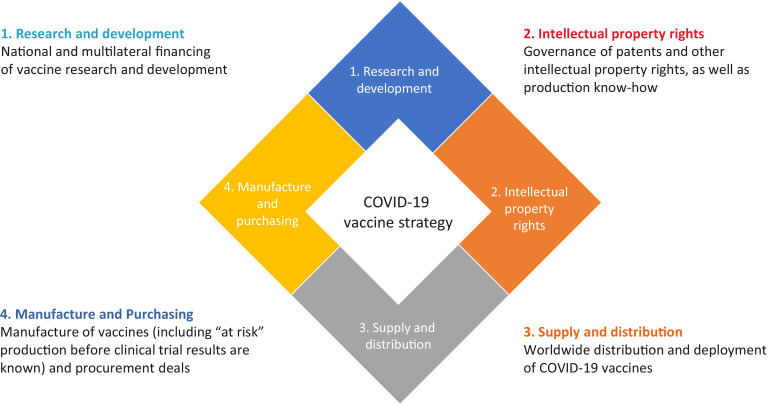
Key elements of national and multilateral COVID-19 vaccine strategies to ensure access to COVID-19 vaccines.

Vaccines have been a key intervention in many PHEICs (including pandemic influenza, polio, Ebola and COVID-19). The speed and inclusivity of their rollout depends on interactions between countries and other actors across several domains, including research and development, supply and distribution, intellectual property rights and manufacture and purchasing (see Figure 1).

***Research and development****.* The COVID-19 pandemic has seen rapid discovery and development of new vaccines, assisted by several factors, including data sharing (such as the genetic sequence of SARS-CoV-2 even before the WHO declaration of a PHEIC) and targeted research funding by governments, acting alone or through multinational bodies.

In this pandemic, the main multinational mechanism to finance vaccine development has been the Coalition of Epidemic Preparedness Innovations (CEPI), a non-profit foundation pooling resources from several national and transnational donors to fund leading COVID-19 vaccine candidates. By pooling resources, CEPI can strike more procurement deals than can most individual countries, thus spreading the risks of failure, mitigating supply chain disruptions, increasing leverage on pricing and distribution, and allocating vaccines to maximise global benefit.

However, bringing new vaccines to market requires substantial funding for research and development [34,35]. Furthermore, multinational efforts such as CEPI have been dwarfed by spending on vaccine development by individual high-income countries acting on their own [36,37]. The most well-known is the US Government's Operation Warp Speed, a public-private partnership to fund COVID-19 diagnostics, vaccines, and therapeutics, which invested well over US$10bn in development of COVID-19 vaccines, therapeutics and diagnostics [38]. In comparison, the EU's Horizon 2020 special COVID-19 grant scheme had awarded under €1 billion through March 2021 for a wide range of research and operational activities including developing vaccines [39]. However, EU-based vaccine research also received support from national funders, so it is unclear whether, in aggregate, the total investment matched the level in the US. What is certain is that EU spending was more diffuse, and less often earmarked for vaccines.

Countries such as the US and UK that acted unilaterally may appear to have been more successful than multilateral efforts, such as those led by the EU or CEPI. However, unilateral vaccine investment is only possible for large, well-resourced countries. Multilateral cooperation has several benefits. Firstly, even for well-resourced countries, backing national champions could create inefficiencies. Second, it allows countries to pool risk, so that an individual country is not dependent on the success of a single candidate it backs. Thirdly, it reduces the dependency of less-resourced countries on the willingness of well-resourced countries to share intellectual property and/or donate vaccine doses after a vaccine is developed and their own populations are vaccinated.

***Manufacture and purchasing.*** In early 2021, vaccine production capacity within the EU was insufficient to meet local demand, let alone demand elsewhere. This was partly the consequence of the EU's relative lack of focus on production in its initial vaccine investment, and insufficiently tight contractual guarantees from producers. To improve cooperation and expedite political decision making, the EU established the Health Emergency Response Authority (HERA) in 2021, mirroring the US's Biomedical Advanced Research and Development Authority (BARDA), which was able to invest quickly and directly in vaccine developers early in the pandemic as part of Operation Warp Speed.

***Supply and distribution****.* Vaccine development is only the first step; vaccines then need to be distributed worldwide. In 2020, global demand for COVID-19 vaccines greatly exceeded supply [40,41]. While the supply situation improved in 2021 [42], the pressure may worsen as additional vaccines are authorized, and countries consider vaccinating younger people and using booster doses or reformulated vaccines to tackle new variants.

Vaccine developers received at least US$10bn in public and non-profit funding [36]. However, funding was not usually tied to international agreements about vaccine distribution. This has resulted in international competition around procurement, inefficient distribution, and even threats to block vaccine supplies from export [43]. This was a wasted opportunity as public funders could have arranged for companies accepting funding to license and share their technologies widely to enable large-scale manufacture and distribution [36]. There is still the opportunity to do this for second generation COVID-19 vaccines as well as in future pandemics.

Given limited supply, high production costs, and vast global demand, market dynamics would dictate that supply flows to countries most able to pay high prices for vaccines, while poorer countries lose out. An alternative would be to set up a multinational agreement to allocate doses according to need, although the optimal way to do so is not straightforward. Most COVID-19-related deaths have been in high-income countries, although the disparity narrows when taking into account under-reporting [44]. However, other considerations besides deaths have been proposed to guide prioritisation, including years of life lost, reducing socioeconomic harms and returning to full functioning [45]. Such considerations would suggest that poorer countries should receive far more doses if allocation was optimal.

However, initiatives to achieve more equitable supply of vaccines have had only limited traction so far. The COVAX Facility is a multilateral initiative designed to procure COVID-19 vaccines on behalf of all participating countries and then supply them to countries in proportion to population, with doses for the poorest countries funded by donors [5]. Although a laudable initiative, COVAX faced shortfalls in funding commitments compared to its target of US$2.6bn [46]. In a supply constrained environment, producers earmarked most doses to high-income countries (including the UK and many EU member-states) able to make financial commitments in advance [47] since COVAX lacked binding mechanisms to enforce principles and rules around global allocation. For example, by June 2021, high-income countries had ordered 6bn vaccine doses, while COVAX had only secured 2.3b [48]. COVAX's inability to procure more doses has forced it to impose commitment agreements on purchasing countries which make it difficult for them to rely exclusively on COVAX for their vaccine supplies; these commitment agreements incentivise national governments to negotiate directly with manufacturers instead of relying solely on COVAX arrangements.

***Intellectual property rights.*** One approach to attain equitable vaccine rollout is to facilitate access to intellectual property and expertise related to COVID-19 vaccines by poorer countries so that the capacity to produce vaccines is more widespread. The United States’ support for a temporary intellectual property waiver for COVID-19 vaccines is a milestone in this endeavour [40]. However, European and other countries with vaccine manufacturing capacity have opposed such an approach on the basis that it will weaken incentives for future drug development, and that regulatory and technical issues are greater barriers than intellectual property. This underscores that patent waivers would need to be accompanied by sharing of technical knowledge to be successful in expanding production capacity.

Several other mechanisms have been proposed to complement or replace intellectual property waivers in facilitating more widespread access to vaccine production capacity. COVAX could purchase the relevant technology and then commission its manufacture on behalf of participating countries, or COVAX could invest in manufacturing capacity expansion similar to grants to manufacturers provided by the EU and US. A more limited option is for vaccine developers to license vaccine candidates for manufacturing by organisations in other countries (such as the agreement between AstraZeneca and the Serum Institute of India [38]), and adjuvants to developers to optimise vaccine manufacturing [49]. Initiatives to reduce bottlenecks in raw materials, harmonise regulatory processes and improve supply chain resilience have also been proposed [50]. Strong multilateral organisations like WHO and the World Trade Organization are needed to provide guidance, allocate tasks, and encourage commitments of solidarity from countries to do their parts for the benefit of the global community.

The alternative to seeking to make vaccines widely available as a global common pool resource is to maximise national self-interest. However, this leads to worse outcomes for everyone. Firstly, it prevents efficiencies in manufacturing, since raw materials and supply chains for vaccine manufacture are global in nature. Few countries are self-reliant for all needed manufacturing inputs [51], let alone the research and development effort needed to bring innovations to populations. Trade barriers around export of vaccines and raw materials for vaccine manufacture have already threatened efficient vaccine production and distribution. Secondly, unequal vaccine access prevents the world economy and trade from recovery. By one estimate, lack of cooperation around COVID-19 vaccine production and distribution could cost up to US$9.2trn in global economic growth [52], with a substantial share borne by high-income countries. It also increases the risk of variants emerging in hotspots that threaten vaccine effectiveness globally [7]. In comparison, the IMF estimates that the cost of vaccinating at least 60% of the population in every country by 2022 would cost $50bn [53]. Yet the current level of funding of COVAX is insufficient to achieve this target.

## 6 Travel policies

Every country imposed some form of travel restrictions in 2020, making them the most extensive travel restrictions in history [54]. The impact of these restrictions on SARS-CoV-2 spread has been mixed. The *cordon sanitaire* imposed on Wuhan in February 2020 was too late to prevent spread to other Chinese cities [55,56]. However, international restrictions probably played a role in delaying spread to countries in early 2020 [55,57]. Several Asia-Pacific countries were able to maintain low COVID-19 incidence throughout 2020 using strong travel restrictions combined with other non-pharmaceutical interventions [58]. Conversely, regions that were slow to impose such restrictions (such as Europe and North America) faced large COVID-19 outbreaks.

WHO was reluctant to support travel restrictions when it declared COVID-19 a PHEIC, having to balance disease mitigation with the social, economic, and legal consequences of these measures, including the flow of supplies needed to mitigate COVID-19 [59]. The travel and tourism sector lost an estimated 143m jobs and $3.8trn in production in 2020 [60]. International Health Regulations dictate that travel restrictions during emergencies should be driven by evidence [55,61]. Evidence suggests that such restrictions are most useful when COVID-19 incidence (and incidence of variants of concern) in destination countries is low; travel restrictions imposed by countries already facing high COVID-19 incidence may have had little effect on epidemic dynamics [62]. However, this principle has not always been applied in practice.

The need for travel restrictions despite their economic cost is especially great when there are highly connected states with dissonant COVID-19 response strategies. Examples include Sweden and Finland [63], and Russia and China [64]. Expedient closing of borders can mitigate asynchronous pandemic waves and variant spread between countries, but within the EU this encounters legal constraints [65]. Furthermore, citizens have been reluctant to accept loss of freedom of movement across the EU, particularly given the constantly changing travel rules. One potential implication for the EU is that free movement brings with it obligations to align health policies. This may be challenging in countries such as Sweden which have pursued different COVID-19 response policies from many of its neighbours. Harmonising vaccine delivery strategies (and not just procurement) may be one area that can achieve greater epidemiological convergence within the EU, and is arguably within its public health competence. A further challenge is that countries differ in terms of economic resilience and reliance on international travel, so regional policy harmonisation may require solidarity mechanisms to support countries economically hardest hit.

On a global level, International Health Regulations may allow evidence-based travel policy coordination that is enshrined in legal commitments to enhance trust between nations. However, these regulations were violated by many of the trade and travel restrictions enacted during the pandemic. There has also been a lack of transparency around how evidence has informed decision making. For instance, the UK's decision in July 2021 to require quarantine from French travellers who were fully vaccinated, could have stemmed from a misreading of GISAID data [66]. Consequently, the WHO has called for stronger legal commitments between nations to enhance international trust [4].

As the pandemic eases in parts of the world, travel instruments such as vaccine or immunity certificates have been proposed, and in some cases, implemented (e.g., the EU Digital COVID Certificate), to support a safe return to cross-border travel [67]. In its present form, the introduction of these instruments has been highly fragmented, with different governments and private sector entities launching separate versions, and no international agreement over inter-operability of record-keeping. WHO has outlined some recommendations to facilitate their inter-operability across countries but opposes making COVID-19 vaccination the sole and mandatory condition for cross-border travel [68]. A further concern is that vaccination may not prevent a traveller from being infected by an immune escape variant. Differing views around the ethical acceptability of vaccine certification may result in political tensions if countries develop independent policies without seeking international consensus [69].

## 7 Conclusion and future steps

The actions of the EU during the pandemic illustrate the tension between short-term nationalistic incentives and long-term imperatives for cooperation towards achieving global public goods. The EU is viewed by some as a credible model for regional cooperation [70], and has shown examples of good governance, including its leadership in developing a pandemic treaty ahead of the next global threat [71]. However, it has struggled to balance preferences of individual member-states (and those of their political leaderships), with the collective interests of all member-states. Such tensions are especially challenging when health care and health policy issues are involved, given how these have hitherto remained largely the responsibility of the member-states. In a pandemic, this can lead to inertia and political indecisiveness at the EU level, with member-states filling the gap with potentially contradictory or competing decisions.

Infectious disease threats will continue to emerge, including new variants of SARS-CoV-2 and other pathogens with pandemic potential. The post-COVID-19 world must overcome the serious setbacks from the pandemic to hard-fought progress in reducing poverty and inequality. Health infrastructure and human resources have been overburdened and will take many years to recover, particularly if governments impose austerity measures as they seek to recover from fiscal expansion during the pandemic.

Strong multilateral collaboration seems necessary for the world to absorb these shocks [2]. Pandemics are opportunities to reimagine governance structures and learn from previous experiences [1]. COVID-19 and previous pandemics have shown the importance of multilateral collaboration in diverse areas, including research and knowledge sharing, discovery, development and distribution of vaccines, and travel restrictions. Beyond infectious diseases, multinational health infrastructure will be vital to tackle the consequences of other global determinants of disease such as climate change and food insecurity. However, as noted above, effective multilateral action is based on certain principles, not all of which may have been fully accepted. Many of the benefits of multilateral collaboration are indivisible and the principle of diffuse reciprocity requires that relationships are based on trust. Yet this has often been in short supply, leading some countries to take a narrowly transactional approach to collaboration, such as the UK's restriction of exports of medicines while benefiting from vaccines manufactured in the EU. It also requires acceptance of mutual responsibility, for example not to pursue policies that allow the spread of the virus to one's neighbours, accompanied by an appropriate dispute settlement process. The absence of such a system has left some governments with no option other than to impose travel restrictions, with the attendant economic consequences.

Our scorecard highlights that multilateral efforts during the COVID-19 pandemic faced challenges such as balancing national and international incentives, as well as developing agile coordination mechanisms that can react rapidly (Table 1). Hence it is important to prepare before emergencies arise, and to develop mechanisms that are robust to political and other unpredictable changes.

Concerted global cooperation is possible, as witnessed by the development of legally binding frameworks like the International Health Regulations. Strengthened governance of multilateral institutions is essential, with mechanisms in which countries contribute according to their ability and obtain support according to their need [72].

Europe is already setting an example. The EU has set out a vision for closer working among its 27 member-states, based on the concept of a European Health Union [73]. One element has been establishing HERA. Although its precise role is as yet uncertain, it may include risk assessment, risk management, and risk communication and will support horizon scanning, technological innovation, and monitoring and support for emergency preparedness [74]. However, it will also have to find ways to collaborate closely with other agencies within the EU, such as the European Medicines Agency (EMA) and the European Centre for Disease Prevention and Control (ECDC), as well as those in the UN system, including the WHO. Meanwhile, the WHO European Region, with its 53 member-states, has established a Pan European Commission on Health and Sustainable Development, which has proposed a comprehensive set of measures at the European and global level, centred on the concept of One Health, that seek to improve the ability to anticipate, prevent, and, where necessary, respond rapidly, including release of financial resources, to a future crisis [75]. Health is also rising up the agenda in regional blocs outside Europe [76] and it seems likely that similar initiatives will emerge elsewhere.

**Search strategy and selection criteria**

To inform this Health Policy article, we conducted a PubMed search for peer-reviewed literature using the key words ((SARS-Cov-2[MeSH Terms]) OR (COVID-19[MeSH Terms])) AND ((cooperation[Title/Abstract]) OR (collaboration[Title/Abstract])). All relevant results until 7 May 2021 were included and no language restrictions were applied. Other relevant articles were identified using a snowballing technique. All descriptions of cooperation between countries, both in response to the COVID-19 pandemic and other emerging infectious disease threats, were considered. Source types were largely restricted to academic publications, although grey literature sources were also included, where relevant. We found 93 papers which met our inclusion criteria and identified 50 with unique themes. Of these, 22 are cited in the main text, while key insights and themes from the remaining 28 are summarised in Supplementary Table 1.

Alt-text: Unlabelled box

## Author contributions

MJ conceived of and designed the manuscript. AA conducted the literature review. All authors had full access to all the data in the manuscript, and were involved in writing and reviewing the manuscript.

## Data availability

All data used are provided in this paper or the accompanying references.

## Declarations of Interest

MJ and PB report grants from the European Union's SC1-PHE-CORONAVIRUS-2020 programme, project number 101003688, during the conducted of the study. MJ reports grants from the National Institute for Health Research (NIHR200929, NIHR200908), during the conduct of the study. PB reports grants from Pfizer, grants from GSK, grants from European Commission IMI, outside the submitted work. AA and YT are employed by HITAP, a research unit in the Thai Ministry of Public Health, which has received funding from the Health Systems Research Institute (HSRI) to conduct a study on “A situational assessment on disease prevention and control for the establishment of a Southeast Asia Center for Infectious Disease Control (SEACID)*"* and “Understanding the challenges to develop monitoring and evaluation framework for COVID-19 vaccination policy in Thailand” and from the National Research Council of Thailand (NRCT) for the initiative "COVID-19 Vaccination Policy Research and Decision Support Initiative in Asia (CORESIA): a regional study on vaccination certificates”. MM declares grants from the European Commission outside the submitted work, is a Commissioner and chair of the Scientific Advisory Board of the Pan European Commission on Health and Sustainable Development, reporting to WHO EURO, and a member of the European Commission's Expert Panel on Effective Ways of Investing in Health. The findings, interpretations and conclusions expressed in this article do not necessarily reflect the views of the funding agencies.

## Appendix Supplementary materials

Image, application 1

## Footnotes

Supplementary material associated with this article can be found, in the online version, at doi:10.1016/j.lanepe.2021.100221.
