# Supplementary material for: Multi-country collaboration in responding to global infectious disease threats: lessons for Europe from the COVID-19 pandemic
Source: Lancet Reg Health Eur. 2021 Oct 7;9:100221. doi: 10.1016/j.lanepe.2021.100221 (PMC8495250; doi:10.1016/j.lanepe.2021.100221)
Supplement: Supplementary file 1 [file mmc1.docx]

**Supplementary appendix to “Multi-country collaboration in responding to global infectious disease threats: lessons for Europe from the COVID-19 pandemic”**

**Supplementary Table 1.** Summary of evidence from existing literature about multi-country cooperation in the COVID-19 response. Only conclusions from the 28 papers identified with unique themes not already mentioned in the main text are summarised here.

| **Domain** | **Studies** | **Key conclusions** |
| --- | --- | --- |
| Research collaboration and information sharing | - Nabavi et al.^1^ - Bernardo et al.^2^ - He et al.^3^ - Kudchadkar et al.^4^ - Malik et al.^5^ - Zhai et al.^6^ - Di Ruggiero et al.^7^ - Harky et al.^8^ - Pai et al.^9^ - Zarocostas et al.^10^ - Venegas-Vera et al.^11^ - Brat et al.^12^ - Mullins et al.^13^ - Vasconcelos et al.^14^ | - There has been a large volume of research output on COVID-19, although knowledge gaps remain. - Clinical collaboration early on in the pandemic have expedited development of treatment and precautionary medical protocols. - International research partnerships have been restricted by the difficulty for individual researchers to identify global collaborators to work with. - High-income countries have traditionally had the greatest research output. However, during the COVID-19 pandemic, countries most impacted by outbreaks, and those less economically affluent have been the highest contributors to global research efforts. - The history of coronaviruses research shows a lack of sustained research activity: there is increased effort following a disease outbreak, but a lull thereafter until the next cycle. |
| Pharmaceutical discovery and development | - Yaya et al.^15^ - Pagliusi et al.^16^ - Holm et al.^17^ | - There has been limited research into therapeutic interventions for COVID-19. - There is urgent need for multi-disciplinary, multi-institutional collaborations. - Low- and middle-income country-based manufacturers play a critical role in this field. Collaboration between them is growing. - Scalable vaccine production requires strong links between academia and industry. - To encourage innovation in vaccine development, assessment of the their value should include socio-economic benefits, so that all the societal returns on investment in vaccine development are fully captured. |
| Vaccine deployment | - Motari et al.^18^ - Phelan et al.^19^ - Shamasunder et al.^20^ | - Many countries have in the past relied on TRIPS flexibilities. The WHO has an opportunity to work at the intersection of WTO mandates and access of health technologies, providing countries with support to recognise their requirements for an effective public health response and draft any legal documentation in a manner that aligns with these needs. - The COVAX Commitment Agreements with countries developed in the second half of 2020 is no longer fit-for-purpose and can be an obstacle for the success of the COVAX Facility. |
| Travel policies | - Medley et al.^21^ - Tran et al.^22^ | - The impact of COVID-19 on tourism is well understood. However, there is also a need to recognise that varying perceptions of risk and the management of local outbreaks in tourist-dependent countries are important determinants of tourism recovery. - Travel restrictions are crucial measures during communicable disease outbreaks, and their role must be understood better. - Travel measures are likely to continue intermittently even as the current pandemic is addressed. - International law is critical to global health and infectious disease mitigation. |
| International politics | - Sebhatu et al.^23^ - Patterson et al.^24^ - Ebrahim et al.^25^ - Collins et al.^26^ - Carroll et al.^27^ - Benis et al.^28^ | - Government policies are influenced by policies initiated in other countries. - Regional bodies have a role in bringing systemic change in health, but they need to address inequalities between member states. - COVID-19 has highlighted shortcomings in country commitment towards international cooperation and global equity agendas. - Global health must consider inequalities in global health power structures. - Integrated One Health (animal, environmental and human) surveillance systems is vital for pandemic preparedness. Low- and middle-income countries need to be incentivised to participate and share information by ensuring their equitable access to technology and capability. |

**References**

1 Nabavi Nouri S, Cohen YA, Madhavan MV, Slomka PJ, Iskandrian AE, Einstein AJ. Preprint manuscripts and servers in the era of coronavirus disease 2019. *J Eval Clin Pr* 2021; **27**: 16–21.

2 Bernardo T, Sobkowich KE, Forrest RO, *et al.* Collaborating in the Time of COVID-19: The Scope and Scale of Innovative Responses to a Global Pandemic. *JMIR Public Health Surveill* 2021; **7**: e25935.

3 He S, Ojo A, Beckman AL, *et al.* The Story of #GetMePPE and GetUsPPE.org to Mobilize Health Care Response to COVID-19 : Rapidly Deploying Digital Tools for Better Health Care. *J Med Internet Res* 2020; **22**: e20469.

4 Kudchadkar SR, Carroll CL. Using Social Media for Rapid Information Dissemination in a Pandemic: #PedsICU and Coronavirus Disease 2019. *Pediatr Crit Care Med* 2020; **21**: e538–46.

5 Malik AA, Butt NS, Bashir MA, Gilani SA. A scientometric analysis on coronaviruses research (1900-2020): Time for a continuous, cooperative and global approach. J Infect Public Health. 2021; **14**: 311–9.

6 Zhai F, Zhai Y, Cong C, *et al.* Research Progress of Coronavirus Based on Bibliometric Analysis. Int J Env. Res Public Health. 2020; **17**. DOI:10.3390/ijerph17113766.

7 Di Ruggiero E, Papadopoulos A, Steinberg M, *et al.* Strengthening collaborations at the public health system-academic interface: a call to action. *Can J Public Health* 2020; **111**: 921–5.

8 Harky A, Mishra V, Ansari DM, Melamed N. Are open-source approaches the most efficient way forward for COVID-19 drug discovery? *Expert Opin Drug Discov* 2021; **16**: 115–7.

9 Pai M. Covidization of research: what are the risks? *Nat Med* 2020; **26**: 1159–1159.

10 Zarocostas J. How to fight an infodemic. *The Lancet* 2020; **395**: 676.

11 Venegas-Vera AV, Colbert GB, Lerma EV. Positive and negative impact of social media in the COVID-19 era. *Rev Cardiovasc Med* 2020; **21**: 561–4.

12 Brat GA, Weber GM, Gehlenborg N, *et al.* International electronic health record-derived COVID-19 clinical course profiles: the 4CE consortium. *Npj Digit Med* 2020; **3**: 109.

13 Mullins E, Hudak ML, Banerjee J, *et al.* Pregnancy and neonatal outcomes of COVID-19: coreporting of common outcomes from PAN-COVID and AAP-SONPM registries. *Ultrasound Obstet Gynecol* 2021; **57**: 573–81.

14 Vasconcelos MH, Alcaro S, Arechavala-Gomeza V, *et al.* Joining European Scientific Forces to Face Pandemics. *Trends Microbiol* 2021; **29**: 92–7.

15 Yaya S, Otu A, Labonté R. Globalisation in the time of COVID-19: repositioning Africa to meet the immediate and remote challenges. Glob. Health. 2020; **16**: 51.

16 Pagliusi S, Hayman B, Jarrett S. Vaccines for a healthy future: 21st DCVMN Annual General Meeting 2020 report. .

17 Holm A. New paths for sustainable solutions to tackle global and emerging infectious threats. *Biologicals* 2020; **65**: 42–5.

18 Motari M, Nikiema J-B, Kasilo OMJ, *et al.* The role of intellectual property rights on access to medicines in the WHO African region: 25 years after the TRIPS agreement. BMC Public Health. 2021; **21**: 490.

19 Phelan AL, Eccleston-Turner M, Rourke M, Maleche A, Wang C. Legal agreements: barriers and enablers to global equitable COVID-19 vaccine access. The Lancet. 2020; **396**: 800–2.

20 Shamasunder S, Holmes SM, Goronga T, *et al.* COVID-19 reveals weak health systems by design: Why we must re-make global health in this historic moment. Glob Public Health. 2020; **15**: 1083–9.

21 Medley AM, Marston BJ, Toda M, *et al.* Use of US Public Health Travel Restrictions during COVID-19 Outbreak on Diamond Princess Ship, Japan, February-April 2020. Emerg Infect Dis. 2021; **27**: 710–8.

22 Tran BL, Chen CC, Tseng WC, Liao SY. Tourism under the Early Phase of COVID-19 in Four APEC Economies: An Estimation with Special Focus on SARS Experiences. Int J Env. Res Public Health. 2020; **17**. DOI:10.3390/ijerph17207543.

23 Sebhatu A, Wennberg K, Arora-Jonsson S, Lindberg SI. Explaining the homogeneous diffusion of COVID-19 nonpharmaceutical interventions across heterogeneous countries. Proc Natl Acad Sci U A. 2020; **117**: 21201–8.

24 Patterson A, Clark MA. COVID-19 and Power in Global Health. Int J Health Policy Manag. 2020; **9**: 429–31.

25 Ebrahim SH, Zhuo J, Gozzer E, *et al.* All Hands on Deck: A synchronized whole-of-world approach for COVID-19 mitigation. *Int J Infect Dis* 2020; **98**: 208–15.

26 Collins FS, Stoffels P. Accelerating COVID-19 Therapeutic Interventions and Vaccines (ACTIV): An Unprecedented Partnership for Unprecedented Times. *JAMA* 2020; **323**: 2455.

27 Carroll D, Morzaria S, Briand S, *et al.* Preventing the next pandemic: the power of a global viral surveillance network. BMJ. 2021; **372**: n485.

28 Benis A, Tamburis O, Chronaki C, Moen A. One Digital Health: A Unified Framework for Future Health Ecosystems. *J Med Internet Res* 2021; **23**: e22189.

29 Thomas DW, Burns J, Audette J, Carroll A, Dow-Hygelund C, Hay M. Clinical development success rates 2006-2015. Washington, D.C.: Biotechnology Innovation Organization, Amplion, and Biomedtracker, 2016.

30 Chit A, Parker J, Halperin SA, Papadimitropoulos M, Krahn M, Grootendorst P. Toward more specific and transparent research and development costs: The case of seasonal influenza vaccines. *Vaccine* 2014; **32**: 3336–40.

31 Davis MM, Butchart AT, Wheeler JRC, Coleman MS, Singer DC, Freed GL. Failure-to-success ratios, transition probabilities and phase lengths for prophylactic vaccines versus other pharmaceuticals in the development pipeline. *Vaccine* 2011; **29**: 9414–6.

32 Hay M, Thomas DW, Craighead JL, Economides C, Rosenthal J. Clinical development success rates for investigational drugs. *Nat Biotechnol* 2014; **32**: 40–51.

33 Pronker ES, Weenen TC, Commandeur H, Claassen EHJHM, Osterhaus ADME. Risk in Vaccine Research and Development Quantified. *PLoS ONE* 2013; **8**. DOI:10.1371/journal.pone.0057755.

34 World Health Organization. An R&D blueprint for action to prevent epidemics: Funding & coordination models for preparedness and response. Geneva, Switzerland, 2016.

35 Wong CH, Siah KW, Lo AW. Estimation of clinical trial success rates and related parameters. *Biostatistics* 2019; **20**: 273–86.
